# The relationships between diabetic male infertility, diabetic cognitive impairment, and the neuroendocrine-immune network: A review

**DOI:** 10.1097/MD.0000000000048631

**Published:** 2026-05-08

**Authors:** Shang-mei Cao, Li Guo, Xue-ya Han, Tian Wang, Wei Wang, Zuo-min Wu, Xiu-hong Fu

**Affiliations:** a Department of Science and Technology Innovation Center, Luohe Central Hospital, The First Affiliated Hospital of Luohe Medical College, Henan Key Laboratory of Fertility Protection and Aristogenesis, Luohe, China; b Pharmacy Department, Luohe Central Hospital, The First Affiliated Hospital of Luohe Medical College, Henan Province Key Laboratory of Traditional Chinese Medicine Formulation and Processing, Henan Province Engineering Research Center for Modernization of Traditional Chinese Medicine Formulation and Clinical Application, Luohe, China.

**Keywords:** cognitive impairment, diabetes, male Infertility, neuroendocrine-immune network

## Abstract

Diabetes mellitus (DM) has become a serious public health problem. Diabetes-related male infertility (DMI) and diabetes-related cognitive impairment (DCI) in DM patients are common and easily ignored problems in clinical practice. To explore the deep-seated connections between DMI, DCI, and DM from the neuroendocrine-immune (NEI) network. We reviewed literature related to DMI and DCI separately, and conducted an in-depth analysis of their relationship with the NEI network. Through an extensive literature review, we found that the important mechanisms involved in DMI and DCI are closely related to the NEI network, including the hypothalamic-pituitary-adrenal-thymus axis and the hypothalamic-pituitary-gonadal-thymus axis. This article elaborates on the different states of these axes in DM patients at different stages, their interrelationships, and how high sugar affects the blood-testis barrier and triggers DMI. The impact of high sugar on the hippocampus leads to a decline in recent memory and cognitive function problems, and the relationship between cognition and the catecholamine system can lead to cognitive impairment during stress states.

## 1. Introduction

Diabetes mellitus (DM) is a heterogeneous multifactorial disease. Globally, around 424 million patients live with DM, and this number is expected to increase rapidly to 552 million by 2030. A multitude of factors have been implicated in the development of DM, and DM has become a serious public health issue.^[1]^ In clinical practice, diabetes-related male infertility (DMI) and diabetes-related cognitive impairment (DCI) caused by DM are quite common, yet their underlying mechanisms remain unclear, and the available literature on this topic is relatively limited. While DM has attracted extensive attention recently, DMI and DCI have not attracted enough clinical attention. To provide a reference for more clinicians regarding these 2 diseases, this study offers a comprehensive discussion and exploration of DMI and DCI.

## 2. Search strategy

A search without any language restrictions was conducted in the MEDLINE, Cochrane Library, and EMBASE electronic databases from inception up to October 2024. The search strategy was adapted to the corresponding database to retrieve relevant studies using keywords and Medical Subject Heading terms “diabetic related cognitive impairment,” “diabetic related male infertility,” “diabetes mellitus,” and their corresponding synonyms and related words/phrases. In this review, we excluded observational studies, animal studies, reviews, editorials, books, and letters to the editor.

## 3. Diabetic male infertility

### 3.1. Epidemiology and pathogenesis of diabetic male infertility

With the increasing prevalence of infertility among individuals of childbearing age globally, infertility has become a significant worldwide concern. Clinically, DMI is common among male patients with DM. Male infertility (MI) refers to the failure of a female to achieve natural conception due to issues with the male after the couple has lived together for more than 12 months and has not used any contraceptives.^[2]^ MI is a common clinical disease. The incidence of infertility ranges from around 11% to 15%, among which about 8% to 22% of infertility cases are caused by male factors.^[3]^ MI is not a disease; it is the collaborative result of many factors. According to a literature review,^[4]^ the macrovascular complications of DM include diabetic nephropathy, diabetic cardiomyopathy, retinopathy, and so on. Impairment of reproductive function is another macrovascular complication of DM.

Various factors underlying reduced fertility due to DM have been reported, including increased DNA damage to sperm, hypothalamic-pituitary-gonadal (HPG) axis function impairment, and advanced glycosylation product disorder, among others. It has also been reported that the hyperglycemic environment in patients with DM damages the blood-testis barrier, impairing the normal microenvironment for germ cells in the testis.^[5]^ However, it remains unclear how the hyperglycemic environment damages the structure and function of the blood-testis barrier. The specific mechanism underlying decreased sperm quality in male patients with DM is also not yet known. At present, the major treatments for MI include antioxidation, L-carnitine, anti-infection, improved microcirculation in the genital system, hormone therapy, and so on. However, these treatments all fail to achieve satisfactory clinical outcomes. There is also a lack of studies on the global symptoms of MI, such as soreness of the waist, debilitation, and sleep disorders.^[6]^

## 4. Diabetic cognitive impairment

### 4.1. Epidemiology and pathogenesis of diabetic cognitive impairment

Cognition is a psychological activity that allows humans to know and understand things. Cognition includes feelings, sensations, imagination, language, memory, and thinking.^[7]^ Mild cognitive impairment (MCI) is a clinical state between normal aging and dementia. Compared with healthy older people, patients with MCI suffer from mild cognitive degeneration, yet their social activities and ability to participate in daily life are not significantly impacted. At present, MCI cannot be adequately explained by medicine or science. MCI is considered to underlie a group of syndromes in DCI.^[8]^ MCI may progress to dementia over time. Research^[9]^ has indicated that patients with type 2 diabetes mellitus (T2DM) account for a significantly high proportion of MCI cases, prompting significant concern about DCI in the medical field. Diabetic encephalopathy (DE) is a major complication of DM that occurs in the central nervous system. DE affects the brain in various ways, such as morphology, electrophysiology, metabolism, neurochemistry, and behavior.^[10]^ Aside from indifference, slow responses, a dull expression, and serious disability of activities of daily living, the clinical symptoms of DE include impairments to memory, judgment, execution, understanding, language application, and visuospatial skills, among others.^[11]^ As independent diseases, “DE” and “DCI” have not yet attracted significant research attention.

Several scholars have reported that patients with T2DM exhibit poor thinking ability, recent memory loss, emotional disorder, decreased sports coordination, or DCI.^[12]^ Transverse and prospective epidemiological studies have demonstrated that T2DM is an independent risk factor for dementia and cognitive impairment without dementia.^[13]^ Globally, China has the largest population of DM patients, with estimates indicating that there are more than 100 million DM patients in China, accounting for about one-third of the patients with DM globally. Over the next several decades, the number of DM patients will continue to increase. Among middle-aged and elderly patients with DM, the prevalence of MCI is relatively high. These MCI patients may go on to develop dementia.^[14]^ DCI intensifies DM, while glucose fluctuation and DM complications intensify MCI. The effects of high glucose levels on cerebral blood flow (CBF) are manifested not only by platelet aggregation dysfunction but also by significantly decreased CBF. High glucose levels can decrease CBF and the cerebrovascular surface area.^[15]^ DM can also influence the blood-brain barrier, as follows: the high glucose level destroys the integrity of the blood-brain barrier; oxidative stress and protein glycosylation occur; and excitatory amino acid toxicity and calcium overload occur.^[16]^ Due to the varied clinical symptoms and lack of specificity, DM can be easily overlooked in the clinical setting. DM-induced MCI can be easily missed and wrongly diagnosed. Based on outpatient clinical trials, the prevalence rate of MCI among middle-aged patients with T2DM is as high as 36%. Another study on the chronic complications of depression and T2DM^[17]^ indicated that 92% of patients with T2DM have MCI, and this rate is even higher among patients aged 50 to 59 years.

### 4.2. Physiological and biochemical basis of learning and memory

Learning refers to the acquisition and development of new experiences.^[18]^ Based on an experimental study of conditioned reflexes, Pavlov argued that the learning process is the process by which a conditioned reflex is established. Memory refers to the maintenance of learned experiences or behaviors,^[19]^ including memorization, consolidation, and reproduction. Memorization is a process in which memory traces of perceived contents are established in the cerebral cortex; this is also known as the acquisition of memory.^[20]^ Consolidation involves the transformation of memory traces from a short-term unstable state to a long-term stable and firm state.^[21]^ Reproduction is the process by which memory traces are recalled or recognized.^[22]^ Learning and memory are extremely complicated physiological and biochemical processes and are influenced by a multitude of factors. Many of the influencing processes and factors are still not fully understood. At present, there are several ideas that are generally accepted.

#### 4.2.1. Central cholinergic nervous system

Clinically, central cholinomimetic drugs are used to improve learning and memory abilities,^[23]^ while anticholinergic drugs show the opposite effect, potentially causing impairments to learning and memory functions. Research has shown that the number of acetylcholine M receptors in brain tissue decreases with increasing age. Further, the amount of acetylcholine in the brain tissue of older patients with dementia is decreased. It is argued that the “cholinergic synapse, also known as the memory synapse, is the physiological basis of learning and memory.” Brain failure among the elderly is related to a decline in central cholinergic nerve function.^[24]^

#### 4.2.2. Central noradrenergic nervous system

Research on the psychopathology of memory deficits has indicated that the contents of norepinephrine (NE) metabolites (e.g., 4-hydroxy-3-methoxyphenylacetic acid) in brain tissues are decreased. Benzedrine can protect from memory deficits since it can promote the release of noradrenaline.^[25]^

#### 4.2.3. Synthesis of brain proteins and energy metabolism

Drugs that can inhibit the synthesis of brain proteins, such as cycloheximide, anisomycin, and puromycin, can all damage memory processes.^[26]^ Nootropic drugs can enhance learning and memory abilities since they can promote the synthesis of brain proteins, increase energy reserves, and resist cerebral injury. Sodium nitrite can trigger hemoglobin degeneration, causing ischemia and hypoxia of brain tissues, thus impairing learning and memory processes. Cerebral vasodilators, such as nimodipine, and some activating blood circulation herbs can improve learning and memory abilities by improving cerebrovascular circulation.

#### 4.2.4. Basic functions of the central nervous system

Excitation and inhibition: Transduction of nerve impulses is accelerated upon excitation, and this facilitates synaptic transmission, which is conducive to the acquisition of information and the consolidation and reproduction of memory. For example, caffeine and small-dose strychnine can stimulate the central nervous system.^[27]^ On the contrary, central inhibitors can inhibit the central nervous system. However, an excessively excited central nervous system will cause dysphoria and impaired learning and memory functions. This can be improved by traditional Chinese medicine, which can calm the nerves.^[28]^

Further, dopamine, 5-hydroxytryptamine (5-HT), and polypeptides such as vasopressin and γ-aminobutyric acid all play close roles in learning and memory functions.^[29]^

## 5. Effects of sex hormones on the cognitive function of mice with DM

Abnormal hormone secretion in the human body is related to cognitive decline.^[30]^ One study reported that, for individuals of the same gender, elderly patients with MCI had lower prolactin, estradiol, and testosterone levels than elderly patients with normal cognition.^[31]^ MCI is caused by decreased activity of multiple sex hormones.^[32]^ It has been reported that androgen can regulate several special cognitive functions; however, the relationship between progesterone and cognitive function is not obvious.^[33]^ Another study reported that the sexual functions of middle-aged and elderly men with MCI often decline, and this can be improved by testosterone supplement therapy.^[34]^ Moreover, testosterone supplement therapy can improve cognitive functions and slow down the development of MCI, thus helping to prevent dementia.^[35]^ An experimental study of the learning and memory abilities of subacute aging mice^[36]^ showed that a normal androgen level has a positive regulatory effect on cognitive functions, with remarkable improvement effects in mice with cognitive decline. Thus, regulation of the sex hormone levels may help control MCI.

Excessively high luteinizing hormone (LH) will cause abnormal activation of mature normal neurons and stimulate physiological and pathological processes (e.g., Aβ proteinosis and excessive phosphorylation of Tau proteins), which will lead to the death of neurons.^[35]^ In another study, the cortical neuron and serum LH contents of inpatients with dementia were found to be significantly increased compared with those of patients of the same age in the control group. Moreover, the severity of dementia is related to the expression intensity of gonadotropin-releasing hormone (GnRH). Reduced expression of GnRH might be one cause of dementia, and this may occur earlier than the influences of estrogen.^[37]^ Sex hormones can improve or delay the occurrence of dementia.^[38]^ However, the regulation of sex hormone levels can cause abnormal blood glucose and blood pressure and can increase the risks of tumors and cardiovascular and cerebrovascular diseases due to difficulties in dosage determination, individual tolerance levels, and adverse reactions. Furthermore, several studies have reported that estrogen or combined estrogen and progesterone supplement therapies can increase the morbidity risks of dementia in postmenopausal women.^[39]^ The unique dual effects of plant estrogens: at low levels, estrogen may combine with the receptor to exhibit effects.^[40]^ However, estrogen is antagonistic to the receptor at high levels. Therefore, the plant estrogen is safer than drug estrogen.

At present, phytoestrogens such as daidzein and many traditional Chinese medicine substitutive hormones are attracting increasing research attention.^[41]^ For example, it has been reported that Icariin, the major active ingredient of *Epimedium* herb, has effects such as plant estrogen.^[42]^ It has been reported that Icariin can improve the learning and memory abilities of fast-aging mice by increasing the serum estradiol level.^[43]^ Alzheimer disease is also referred to as “type-3 diabetic mellitus” since it has been found that Alzheimer disease represents a kind of neuroendocrine anomaly.^[44]^ Patients with T2DM often have sex hormone disorders related to insulin resistance and insulin sensitivity.^[45]^ Patients with DM have insufficient insulin. In this case, too many sex hormones become the antagonist of insulin, thus intensifying the existing glucose metabolic disorder.^[46]^ The metabolic disorders and gonadal dysfunction secondary to DM interact with each other, causing or exacerbating complications of DM.^[47]^

## 6. Neuroendocrine-immune mechanism involved in DMI and DCI

Research indicates that the mechanisms involved in DMI and DCI are closely related to the neuroendocrine-immune (NEI) network. The NEI network covers the hypothalamic-pituitary-adrenal-thymus axis (HPAT)^[48]^ and the hypothalamic-pituitary-gonadal-thymus axis (HPGT).

The feedback regulation of the HPAT axis involves the following steps.^[49]^ The adrenal cortex secretes excessively high levels of cortisol and corticosterone. This inhibits the secretion of hypothalamic corticotropin-releasing hormone. This, in turn, inhibits the secretion of pituitary adrenocorticotropic hormone (ACTH). As a result, ACTH declines. The low level of circulating cortical hormone stimulates the activity of various mature lymphocytes and accelerates the development of immature prolymphocytes into effector lymphocytes. The low level of cortical hormone also increases the secretion of thymic hormone, which can influence the maturity of lymphocytes. The high level of thymic hormone increases glucocorticoids (GCS) through positive feedback regulation of the hypothalamus and pituitary gland, thereby stimulating interleukin 1 and glucocorticoid factor secreted by lymphocytes and monocytes. Interleukin 1 and glucocorticoid factor act on the hypothalamus and pituitary gland, respectively, to increase GCS.

### 6.1. Changes to the HPAT axis in patients with DM

It has been reported that patients with early-stage DM exhibit increased secretion of cortisol,^[50]^ indicating hypothalamic-pituitary-adrenal (HPA) axis dysfunction. HPA axis dysfunction might be the possible mechanism underlying the high cortisol levels in patients with DM. Such a mechanism may be related to the following factors. First, chronic hyperglycemic stimulation in patients with DM. The hippocampus is not only the micturition center for negative feedback regulation of the HPA axis but also a region that is sensitive to stress damage.^[51]^ It participates in inhibitory regulation of the stress response of the HPA axis.^[52]^ Hyperglycemia is related to both inflammation markers and neuropathic automatic stability regulation. Stress and the autonomic nervous system play an important role in the occurrence and development of DM.^[53]^ Second, the occurrence of chronic oxidative stress in patients with DM can damage the hippocampus and cause degenerative changes in the hippocampus.^[54]^ In this way, the inhibitory effect of the hippocampus over the HPA axis is weakened.^[55]^ HPA hyperfunction can increase the secretion of thymic hormone and strengthen the immune system of the body.^[56]^

Patients with DM also might suffer from relative adrenal insufficiency.^[57]^ Due to the long-term hyperglycemic stimulation, the adrenal cortex may fail to meet the needs of the body under a stress state; this manifests as relative adrenocortical hypofunction. Since patients with DM are in a chronic stress state of hyperglycemia, the HPA axis might become fatigued and weak in response to stimuli.^[58]^ Hence, the immune system of the body will be weakened.^[59]^ Additionally, hyperglycemia, along with concurrent dyslipidemia or hypertension, may affect the normal function of cerebral blood vessels and cause cognitive decline.

To sum up, the state of the HPAT axis in patients with DM varies as a function of the disease stage. It is hyperactive in the early disease stage and then becomes degenerated in the chronic stage.

### 6.2. Changes to the HPGT axis in patients with DM

The HPG axis is also an important component of the NEI network. There is sufficient evidence to suggest that hyperirritability or disorder of the HPA axis is closely related to stress hypofunction of the HPG axis. GCS, which is secreted during activation of the HPA axis in the early stage of DM, can also inhibit the gonadotropin function of the pituitary gland. This is also closely related to stress inhibition of the HPG axis and reproductive endocrine disorders.^[60]^ Based on the above studies, activation of the HPA axis can affect the HPG axis. However, the development and progression mechanisms require further study.

The HPG axis can also regulate immunity.^[61]^ The hypothalamus can release GnRH to act on the pre-pituitary gland to facilitate the release of follicle-stimulating hormone and LH. These, in turn, act on the gonads through the blood, thus facilitating the synthesis and release of sex hormones and participating in immunoregulation. One study reported that estrogen can regulate immunity. Estrogen was found to promote the immunity of female animals under a physiological dose but inhibit immunity under a high dose. Thus, the effects of androgens on immunity are like a double-edged sword. To sum up, the HPGT axis can not only regulate the reproductive endocrine functions of the body but is also an important component of the NEI network, which regulates immunity in the body.

Research indicates that patients with DM are struggling with metabolic and hemodynamic disorders, renal hyperfiltration, renin-angiotensin-aldosterone, and endocrine disorders, which influence the normal rhythms of the HPAT axis. Moreover, hyperglycemic stimulation can damage the hippocampus and cause degeneration. Hence, the inhibitory effect of the hippocampus over the HPAT axis is weakened. Adrenocortical hyperfunction in patients with DM may weaken or eliminate the valleys in the day-night variation rhythm of the HPAT axis and increase the GCS level in plasma, which, in turn, will directly destroy the hippocampus.^[62]^ Meanwhile, short-term memory may decline, and cognitive function will become impaired. The hyperirritability or disorder of the HPAT axis further brings stress hypofunction of the HPGT axis and decreases the secretion of sex hormones, thus influencing the immune system.

Another possible mechanism by which individuals are more sensitive to DCI under a stress state is related to NE in the catecholamine system. NE has a regulatory effect on the brain through α1, α2, and β receptors. Generally speaking, the activity of the α2 receptor in the brain is related to the maintenance of normal cognitive functions. Continuous and excessive activation of the α1 receptor might cause cognitive disorders.^[63]^ Under a normal alert state, there is an appropriate amount of NE in brain cells, and the α2 receptor plays a dominant role, thus maintaining normal cognitive functions. Abundant NE is produced under stress states, and the α2 receptor plays a dominant role.^[64]^ This might be one of the mechanisms underlying the long-term stress state suffered by patients with DCI.

### 6.3. Key neurotransmitters in the NEI network

As a neurotransmitter, 5-HT is mainly distributed in the pineal body and hypothalamus.^[65]^ Under the influence of stimuli, 5-HT is released from particles and dispersed into the blood. It is then captured by and stored in blood platelets. The 5-HT neuron system acts widely on the prefrontal cortex, limbic system, pituitary activity, and sexual behaviors. It is an important neurotransmitter involved in the regulation of cognitive and emotional stability.^[66]^ Dopamine is a critical neurotransmitter in the hypothalamus and pituitary gland.^[67]^ The dopamine neuron system plays a crucial role in pleasure, reward, addiction, and behavioral motivations. NE neurons are located in the lower brain stem,^[68]^ especially in the mesencephalic reticular formation, locus coeruleus of the pons, and ventrolateral part of the medullary reticular formation.^[69]^ The catecholamine system is closely related to the maintenance of cerebral functions.^[70]^

### 6.4. Other substances that influence cognition and the NEI network

Homocysteine (Hcy) is a sulfhydryl-carrying amino acid; it is mainly derived from methionine in food. Hcy is an important intermediate product of the metabolic process of methionine and cysteine. However, it does not participate in the synthesis of proteins in the body. There are 2 metabolic pathways of Hcy in the body. One transforms Hcy into methionine through methylation. The other transforms Hcy into cysteine and α-ketobutyrate through irreversible trans-sulfurization.^[71]^ More than 90% of Hcy is discharged through the kidneys. The body has an extremely low content of Hcy, usually lower than 15 µmol/L. Excessively high content of Hcy will cause damage to the body, including renal and urinary system damage, cerebrovascular damage, and cognitive impairment. High Hcy during pregnancy may even cause fetal malformations.

Aldosterone (ALD) is a hormone that regulates the blood volume in the human body.^[72]^ It maintains the water-salt balance by regulating renal reabsorption of Na^+^. ALD can also regulate the extracellular fluid volume and electrolytes. When the extracellular fluid volume increases, ALD secretion decreases, resulting in decreased renal reabsorption of Na^+^ and water and a decreased extracellular fluid volume. A decreased blood Na^+^ level and an increased serum potassium level can also stimulate ACTH to increase the secretion of ALD. The mechanism underlying ALD-induced cognitive impairment might involve renin-angiotensin-aldosterone activation. It may cause apoptosis of nerve cells, white matter damage, and synaptogenesis inhibition by inducing ROS activation, pro-inflammatory factor release, continuous activation of the HPA axis, and glucose metabolic disorder, among other potential mechanisms.

## 7. NEI mechanisms linking DMI and DCI

The damage of high glucose to the hippocampus can cause hippocampal lesions, leading to weakened inhibition of the HPAT axis in the hippocampus. Adrenal cortex dysfunction in DM patients can disrupt the HPAT axis, causing an increase in plasma GCS levels and exacerbating hippocampal damage. Hippocampal injury can lead to cognitive dysfunction and trigger DCI. The main mechanism of DMI is that the high glucose environment in the patient’s body can disrupt the blood-testis barrier, causing the reproductive cells in the testes to lose their normal microenvironment. Testicular supporting cells are an important component of the blood-testis barrier. High glucose damages the stability of the blood-testis barrier by damaging the tight junction proteins on supporting cells, leading to a decrease in male sperm quality and the failure of assisted reproductive technology.

Immune factors are a common mechanism involved in the HPAT axis and HPGT axis in the NEI network system. In different stages of DM, the HPAT axis in the body is in different states, with early manifestations of hyperactivity and chronic manifestations of decline. Hyperactivity or disorder of the HPAT axis leads to decreased stress function of the HPGT axis, decreased secretion of sex hormones, and thus affects the reproductive and immune systems. In addition, another possible mechanism by which individuals are more sensitive to DCI under a stress state is related to NE in the catecholamine system. NE has a regulatory effect on the brain through α1, α2, and β receptors. Generally speaking, the activity of the α2 receptor in the brain is related to the maintenance of normal cognitive functions. Continuous and excessive activation of the α1 receptor might cause cognitive disorders. Under a normal alert state, there is an appropriate amount of NE in brain cells, and the α2 receptor plays a dominant role, thus maintaining normal cognitive functions. Abundant NE is produced under stress states, and the α2 receptor plays a dominant role. This might be one of the mechanisms underlying the long-term stress state suffered by patients with DCI.

## 8. Clinical and therapeutic implications

The mechanism studies of the NEI network system listed in this article have certain guiding significance for clinical practice. Many hospitals, including Xuanwu Hospital, Capital Medical University, have conducted clinical research on dementia patients involving the NEI network system. It can be seen that this conclusion has attracted the attention of clinical doctors and researchers. We also hope that the publication of this article will draw increasing attention from more people to this conclusion and promote further research in this area.

### 8.1. Limitations

There are a few disease-related literature discussed in this paper, and their quality is generally average. In the future, if new perspectives emerge or higher-quality papers are published, more discussions on these 2 issues can be conducted.

## 9. Conclusion

The brain controls growth, development, and reproduction, including the growth and development of neurons. Hormone regulation is crucial to the growth and development of neurons. Neurotransmitters and hormones are important participants in the NEI network. Immune factors are the common mechanism of the HPAT axis and HPGT axis, which participate in the NEI network system. The state of the HPAT axis in patients with DM varies as a function of the disease stage. It is hyperactive in the early disease stage and then becomes degenerated in the chronic stage.

## Author contributions

**Conceptualization:** Shang-mei Cao, Xue-ya Han.

**Investigation:** Shang-mei Cao, Tian Wang.

**Methodology:** Shang-mei Cao.

**Project administration:** Wei Wang.

**Funding acquisition:** Zuo-min Wu.

**Supervision:** Xiu-hong Fu.

**Writing – original draft:** Shang-mei Cao, Zuo-min Wu.

**Writing – review & editing:** Shang-mei Cao, Li Guo, Xue-ya Han, Xiu-hong Fu.
